# Intubation in acute alcohol intoxications at the emergency department

**DOI:** 10.1186/s13049-020-0707-2

**Published:** 2020-02-10

**Authors:** Thomas C. Sauter, Katharina Rönz, Trevor Hirschi, Beat Lehmann, Christopher Hütt, Aristomenis K. Exadaktylos, Martin Müller

**Affiliations:** 1 Department of Emergency Medicine, Inselspital, Bern University Hospital, University of Bern, Freiburgstrasse 8, 3010 Bern, Switzerland; 2 Medical Skills Lab, Charité Medical School Berlin, Berlin, Germany; 3 Department of Anaesthesiology, Hôpital Cantonal Fribourg, Fribourg, Switzerland; 4 Department of Anaesthesiology, Inselspital, Bern University Hospital, University of Bern, Bern, Switzerland; 5 Department of Anaesthesiology and Intensive Care, University Hospital Bonn, Bonn, Germany

**Keywords:** Alcoholic intoxication, Drug overdoses, Unconsciousness, Glasgow coma scale, Intratracheal intubation, Airway management

## Abstract

**Background:**

Guidelines recommend endotracheal intubation in trauma patients with a Glasgow coma scale (GCS) < 9 because of the loss of airway reflexes and consequential risk of airway obstruction. However, in patients with acute alcohol intoxication guidelines are not clear. Thus, we aimed to determine the proportional incidence of intubation in alcohol intoxication and compare the clinical characteristics of intubated and non-intubated patients, as well as reasons for intubation in all patients and in the subgroup of patients with reduced GCS (< 9) but without traumatic brain injury.

**Methods:**

We performed a retrospective analysis of all consultations to an urban ED in Switzerland that presented with an acute alcohol intoxication between 1st June 2012 and 31th Mai 2017. Patient and emergency consultations’ characteristics, related injuries, intubation and reason for intubations were extracted. As a subgroup analysis, we analysed the patients with a GCS < 9 without a traumatic brain injury.

**Results:**

Of 3003 consultations included from 01.06.2012 to 31.05.2017, 68 were intubated, leading to a proportional incidence of 2.3% intubations in alcohol-intoxication. Intubated patients had a lower blood alcohol concentration (1.3 g/kg [IQR 1.0–2.2] vs. 1.6 g/kg [IQR1.1–2.2], *p* = 0.034) and less often suffered from chronic alcohol abuse (1183 [39.4%] patients vs. 14 [20.6%], *p* = 0.001) than non-intubated patients. Patients with trauma were intubated more often (33 patients [48.5%] vs. 742 [25.3%], *p* < 0.001). In subgroup analysis, 110/145 patients (74.3%) were not intubated; again, more intubated patients had a history of trauma (9 patients [25.7%] vs. 10 [9.1%], *p* = 0.011).

**Conclusions:**

Intubation in alcohol-intoxicated patients is rare and, among intoxicated patients with GCS < 9, more than two thirds were not intubated in our study - without severe complications. Trauma in general, independent of the history of a traumatic brain injury, and a missing history of chronic alcohol abuse are associated with intubation, but not with blood alcohol concentration. Special caution is required for intoxicated patients with trauma or other additional intoxications or diseases.

## Introduction

The incidence of self-intoxication is high in preclinical or clinical emergency medicine is high and this is mostly alcohol poisoning. In a Swiss study, 3.3% of all non-traumatic emergency department patients in 2007 were diagnosed with alcohol intoxication [1]. It is essential that airway management should be considered in the clinical treatment of overdosed individuals. When deciding whether to perform endotracheal intubation in a non-traumatic patient, it is essential to consider both the impaired state of consciousness and respiratory failure [2]. A common approach in evaluating the consciousness of the intoxicated patient is to assess patients by the Glasgow Coma Scale (GCS) [3, 4]. Although there are unambiguous recommendations on the intubation of patients with cerebral trauma (GCS < 9) [5], the guidelines for intoxicated patients are far less clear. This is also due to differences in pathophysiology in patients with or without a cerebral injury. In intoxicated coma patients, intubation is less often needed to prevent hypoxaemia and hypercapnia by controlling the airways and ventilation, but is still crucial in preventing tracheal aspiration [6]. Furthermore, there is a clear trend away from the use of GCS assessment for airway management of neurological emergencies. Emergency neurological life support guidelines do not mention GCS scoring for patients with an “airway at risk” [2].

Studies on the correlation of GCS and airway reflexes - such as gagging and coughing - do not support decisions for endotracheal intubation that are based on mere scores [7–9]. Accordingly, some published studies promote early GCS-centred intubation in the intoxicated patient population [10, 11], while others studies have reported positive outcomes with rather liberal airway management regimes for the non-trauma comatose patient [12, 13].

The aims of this retrospective study of patients with acute alcohol intoxications were i) to determine the proportional incidence of intubation in alcohol intoxication, ii) to compare clinical characteristics of intubated and non-intubated patients in all patients and in the subgroup of patients with reduced GCS (< 9) but without clinical evidence of traumatic brain injury, and iii) to explore reasons for intubation for all patients and in the subgroup detailed above.

## Methods

This retrospective analysis covered the study period of five years (1st June 2012 to 31^th^ May 2017) and included all consultations of patients aged at least 16 years who were diagnosed with acute alcohol intoxication as a primary or secondary diagnosis at our ED of Bern University Hospital (Switzerland). The general characteristics of the population served by our hospital are described elsewhere [14].

As the diagnosis of acute alcohol intoxication is based on clinical findings as well as on patient history on admission [15], we used the reported diagnosis of the treating physician to identify acute alcohol intoxication. Patients were identified using a four step approach: Firstly, a full text keyword-search was performed in the medical report using “alcohol intoxication” and “mixed intoxication” with different semantic combinations. Secondly, duplicates with the different search keywords were removed. Thirdly, an experienced clinician (TH) screened the discharge diagnosis manually to identify acute alcohol intoxication as a primary or secondary diagnosis (e.g. excluding patients with a past diagnosis of alcohol intoxication only). Fourthly, the medical records of the remaining consultations were screened in full text (KR) as a quality control to ensure the validity of the diagnosis screening.

### Parameter extraction

Data such as i) demographic data i.e. sex, nationality, ii) consultation characteristics i.e. triage and shock room use as markers for “initial impression of urgency”, leading discipline, hospital admission as well as iii) laboratory variables i.e. sodium, potassium, urea, glucose, and osmolality were extracted automatically from the patient database. To estimate the blood alcohol concentration in g per kg in full blood, we used the formula published by Lynd et al. [16]. If no adequate laboratory data were available, we used the alcohol concentration determined by a breath alcohol test (Lion alcolmeter® 500, Lion Laboratories Limited), as an approximation for the blood alcohol concentration. The initial triage at our ED is routinely performed for every patient by specially trained nurses according to the Swiss triage Scale [16].

The following parameters were extracted through manual coding of the full ED report (KR, TH): i) consultation characteristics i.e. type of admission, presence and type of mixed substance intoxication (medical or illicit drug in addition to alcohol), GCS [3], suicidal intent, aggressive patient, history of chronic alcohol consumption, necessity of forced treatment, such as detention by the police or administration of sedative medication under restraint due to the risk of foreign or self-injury during intoxication, police attendance, psychiatric involvement and admission; ii) concomitant injuries e.g. trauma, traumatic brain injury, fractures; iii) treatment such as sutures, emergency surgery, endotracheal intubation including details and reason for intubation. The decision to intubate was made by the treating physician. The extracted reasons for intubation are grouped to allow comparison. Only endotracheal intubations were included, but not supraglottic airway devices. Rapid sequence induction is standard care in our population for emergency inductions and did not change during the study period.

The documented comorbidities in the diagnosis list and specific drug intake were determined through a validated full text parser of the ED medical report; see Additional file 1: Table S1. The following intake of drugs was determined as defined by ATC classes [17]: antidiabetics (ATC code A10), antithrombotics (B01), antihypertensives (C02, C04-C09), diuretics (C03), opioids (N02A), and antiepileptics (N03).

### Ethical considerations

The study was approved by the regional ethics committee of the Canton of Bern, Switzerland. Patients who refused to give general consent for the use of their anonymised data were excluded from the study (KEK: 3841). Subsequent withdrawal of the general consent is also possible at any time and such a patient would be excluded. The need for informed consent was waived by the ethics committee for the present study.

### Statistical analysis

We used Stata® 13.1 (StataCorp, The College Station, Texas, USA) to perform the analysis of the patients.

The Shapiro-Wilk test was performed to test for normality of continuous data (i.e. age, blood alcohol concentration and GCS). Normal distributed continuous data are presented with mean (standard deviation), non-normally distributed data with median accompanied by the interquartile range (IQR). For the GCS, median and mean are presented to illustrate the distribution.

The distribution of categorical variables was described using the absolute values accompanied by the relative number in the group in each stratum.

The difference between intubated and non-intubated patients was tested using a chi-square test (categorical variables) or the Wilcoxon rank sum test (continuous data). The significance level was set to *p* < 0.05.

## Results

**Fig. 1:**
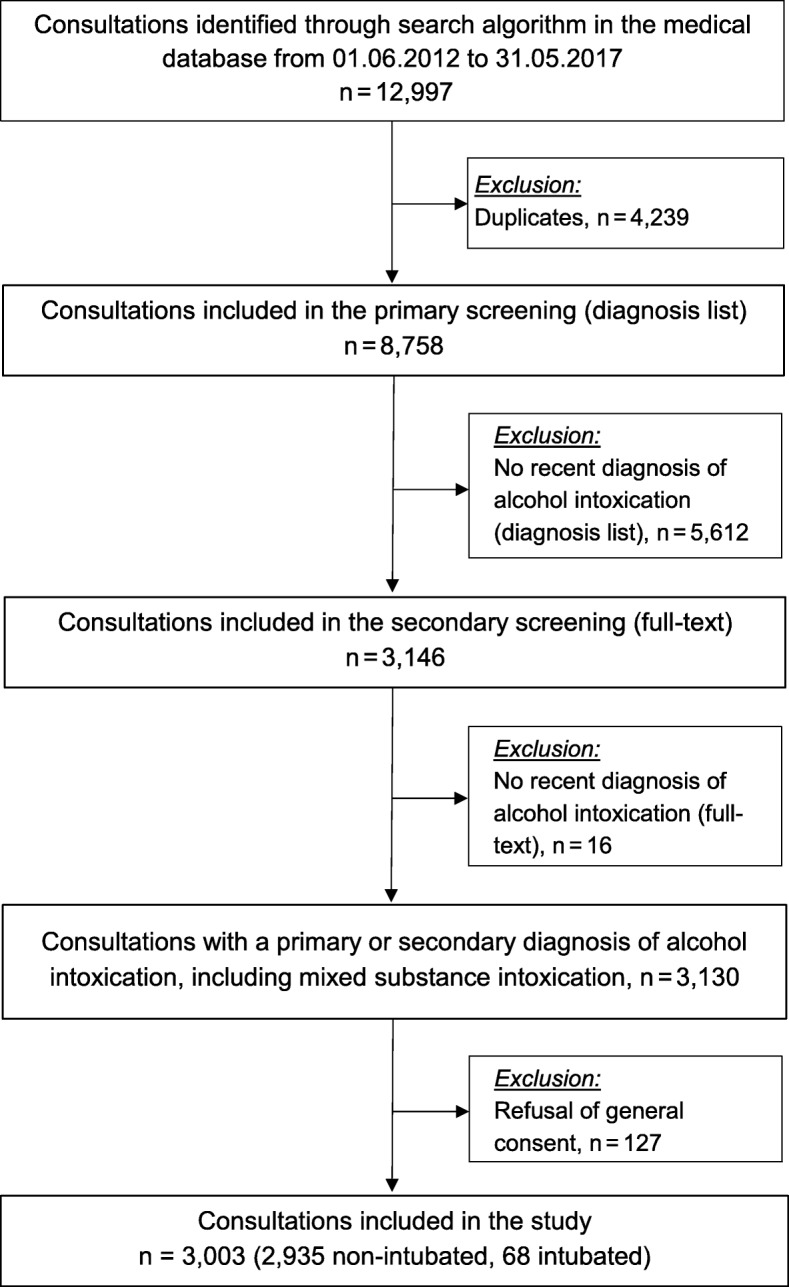
Flowchart of the study

We identified 12,997 patients through the search algorithm in the medical database from ^1st^ June 2012 to 31th May 2017 (Fig. 1). Of those, we excluded 4239 duplicates. The remaining 8758 consultations were manually screened in the diagnosis field. When there was no documented recent diagnosis of alcohol intoxication, we excluded 5612 consultations (e.g. history of alcohol intoxication only). Finally, the remaining 3146 consultations were screened in full text and 16 consultations were excluded as no primary or secondary diagnosis of acute alcohol intoxication was found. In 127 consultations, the patient refused the general consent and was therefore excluded. Thus, 3003 consultations suited the eligibility criteria and were included in the study.

Of all 3003 consultations, 68 were intubated - leading to a proportional incidence of 2.3 intubations in 100 consultations with alcohol intoxication.

### Patient characteristics of all patients with alcohol intoxication

The characteristics of all 3003 included consultations grouped by the airway management - non-intubated (*n* = 2935) vs. intubated (*n* = 68) - are shown in Table 1.

**Table 1:** Consultation characteristics of all intubated (n = 68) and non-intubated patient consultations with alcohol intoxication (n = 2935)

	Total (*n* = 3003)		Intubation (n = 68)		No Intubation (n = 2935)		p
Sex, [n (%)]							
Male	1899	(63.2)	50	(73.5)	1849	(63.0)	0.075
Age, [median (IQR)]	39.0	(27–52)	42.0	(28–53)	39.0	(27–52)	0.435
Personal history, [n (%)]							
Diabetes mellitus	135	(4.5)	2	(2.9)	133	(4.5)	0.530
Liver disease	191	(6.4)	3	(4.4)	188	(6.4)	0.503
Dementia	22	(0.7)	0	(0.0)	22	(0.8)	0.473
Myocardial infarction	81	(2.7)	2	(2.9)	79	(2.7)	0.902
Malignancy	59	(2.0)	2	(2.9)	57	(1.9)	0.559
Chronic kidney disease	12	(0.4)	1	(1.5)	11	(0.4)	0.157
Medication therapy on admission, [n (%)]							
Any medication	619	(20.7)	13	(19.1)	606	(20.7)	0.752
Antiepileptic	216	(7.2)	4	(5.9)	212	(7.2)	0.669
Opioids	84	(2.8)	4	(5.9)	80	(2.7)	0.120
Diuretic	83	(2.8)	1	(1.5)	82	(2.8)	0.509
Antihypertensive	286	(9.5)	6	(8.8)	280	(9.6)	0.838
Antithrombotic	207	(6.9)	6	(8.8)	201	(6.9)	0.528
Antidiabetic	105	(3.5)	1	(1.5)	104	(3.6)	0.356
Type of admission, [n (%)]							
Ambulance	1827	(60.9)	43	(64.2)	1784	(60.8)	
General Practitioner	29	(1.0)	0	(0.0)	29	(1.0)	
External Hospital	110	(3.7)	11	(16.4)	99	(3.4)	
Police	399	(13.3)	0	(0.0)	399	(13.6)	
Air Rescue	38	(1.3)	11	(16.4)	27	(0.9)	
Walk-In	598	(19.9)	2	(3.0)	596	(20.3)	< 0.001
Triage, [n (%)]							
Life-threatening	238	(7.9)	42	(61.8)	196	(6.7)	
Urgent conditions	1133	(37.7)	16	(23.5)	1117	(38.1)	
Semi-urgent conditions	1457	(48.5)	6	(8.8)	1451	(49.4)	
Non-urgent conditions	100	(3.3)	0	(0.0)	100	(3.4)	
Missing	75	(2.5)	4	(5.9)	71	(2.4)	< 0.001
Primary shock room use, [n (%)]	270	(9.0)	62	(91.2)	208	(7.1)	< 0.001

There was no significant difference in age between the intubated and non-intubated patients (non-intubated: 39 years, IQR 27–52 vs. intubated: 42 years, IQR 28–53, *p* = 0.435). Nearly two thirds of all patients were male (63.2%), without a significant difference between the airway management groups (*p* = 0.075).

Variables that were significantly different in intubated vs. no intubated consultations (*p* < 0.001) were the type of admission, the triage and primary shock room use. The predominant admission type was via ambulance (60.9% in total and 64.2% of all intubated patients). Of all patients with the most urgent triage category of a life-threatening situation (*n* = 238), 42 (61.8%) were intubated. In almost half of the consultations, a psychiatrist was involved (*n* = 1327, 44.2%). In total, 1143 (38.1%) of the patients were admitted to the ward for further treatment and surveillance.

### Associations with intubation

The characteristics of the intoxication that were associated with intubation were a lower GCS (*p* < 0.001), the presence of mixed intoxication (*p* = 0.025) or lower blood alcohol concentration (*p* = 0.034), the absence of a history of chronic alcohol consumption (*p* = 0.001) or police attendance (p = 0.001), as well as signs and diagnosis of trauma and concomitant injuries such as traumatic brain injury, cerebral bleeding, fractures, and luxations (all p < 0.001); see Table 2.

**Table 2:** Association of intoxication characteristics, clinical presentation, concomitant injuries, and treatment with intubation

	Total (*n* = 3003)		Intubation (*n* = 68)		No Intubation (*n* = 2935)		p
Intoxication characteristics							
Mixed intoxication, [n (%)]	1029	(34.3)	32	(47.1)	997	(34.0)	0.025
Medical drug mixed intoxication, [n (%)]	664	(22.1)	27	(39.7)	637	(21.7)	< 0.001
Illicit drug mixed intoxication, [n (%)]	535	(17.8)	10	(14.7)	525	(17.9)	0.498
GCS, [median (IQR)]	15.0	(14–15)	3.0	(3–7.5)	15.0	(14–15)	< 0.001
GCS (< 9), [n (%)]	173	(5.8)	53	(77.9)	120	(4.1)	< 0.001
Blood alcohol concentration, g/Kg [median (IQR)]^a^	1.6	(1.1–2.2)	1.3	(1.0–2.2)	1.6	(1.1–2.2)	0.034
Suicidal intent, [n (%)]	350	(11.7)	8	(11.8)	342	(11.7)	0.977
Aggression, [n (%)]	230	(7.7)	4	(5.9)	226	(7.7)	0.577
History of chronic alcohol consumption, [n (%)]	1183	(39.4)	14	(20.6)	1169	(39.8)	0.001
Forced treatment, [n (%)]	27	(0.9)	1	(1.5)	26	(0.9)	0.614
Police attendance, [n (%)]	811	(27.0)	6	(8.8)	805	(27.4)	0.001
Concomitant Injuries/disease							
Trauma, [n (%)]	775	(25.8)	33	(48.5)	742	(25.3)	< 0.001
Traumatic brain injury, [n (%)]	203	(6.8)	20	(29.4)	183	(6.2)	< 0.001
Superficial wound, [n (%)]	399	(13.3)	9	(13.2)	390	(13.3)	0.990
Contusion, [n (%)]	190	(6.3)	7	(10.3)	183	(6.2)	0.174
Luxation, [n (%)]	25	(0.8)	4	(5.9)	21	(0.7)	< 0.001
Fractures, [n (%)]	246	(8.2)	25	(36.8)	221	(7.5)	< 0.001
Cerebral bleeding, [n (%)]	53	(1.8)	11	(16.2)	42	(1.4)	< 0.001
Treatment and Outcome							
Psychiatrist involvement, [n (%)]	1327	(44.2)	0	(0.0)	1327	(45.2)	< 0.001
Sutures, [n (%)]	356	(11.9)	7	(10.3)	349	(11.9)	0.687
Emergency surgery, [n (%)]	71	(2.4)	5	(7.4)	66	(2.2)	0.006
Hospital admission, [n (%)]	1143	(38.1)	65	(95.6)	1078	(36.7)	< 0.001
Psychiatry admission, [n (%)]	726	(24.2)	0	(0.0)	726	(24.7)	< 0.001

^a^data available in 76.7% (*n* = 2303) of the patients

Of all patients admitted to hospital, 63.5% were admitted to the psychiatric ward. Patients with chronic alcohol dependence and the desire for withdrawal are admitted to psychiatry on an inpatient basis in our setting as well as patients who represent a potential danger to themselves or others are admitted to psychiatry for acute therapy.

### Reasons for intubation in all patients with alcohol intoxication

Of the intubated patients, 21 (30.9%) needed a secured airway before arriving at the hospital; the remaining 47 (69.1%) were intubated after arriving in the hospital. No patient needed to be intubated during emergency surveillance after the initial assessment. The reasons for intubation are summarised in Table 3.

**Table 3:** Reasons for intubation in patients with alcohol intoxication (*n* = 68)

Reason, [n (%)]	Total		Prehospital intubation		In-hospital Intubation	
Traumatic brain injury and GCS < 9	9	(13.2)	2	(2.9)	7	(10.3)
Failure to oxygenate/ ventilate with non-invasive methods	6	(8.8)	5	(7.5)	1	(1.5)
Protecting airway (vomiting)	6	(8.8)	2	(2.9)	4	(5.9)
Protecting airway (facial trauma/ blood)	5	(7.5)	2	(2.9)	3	(4.4)
Cardiac arrest/CPR	3	(4.4)	2	(2.9)	1	(1.5)
Agitation/pain	11	(16.2)	2	(2.9)	9	(13.2)
Shock	1	(1.5)	0	(0)	1	(1.5)
Surgical procedure	3	(4.4)	1	(1.5)	2	(2.9)
GCS < 9 without additional specific reason being documented	24	(35.2)	5	(7.5)	19	(27.9)

A GCS below nine was found in 173 (5.8%) consultations. This corresponded to 53 (77.9%) of all 68 intubated patients, so that GCS < 9 was the main reason for intubation (with traumatic brain injury (*n* = 9) or without brain injury (*n* = 24)).

Six patients (8.8%) needed intubation because of insufficient oxygenation or ventilation. Eleven patients (16.2%) were intubated to protect the airway from vomit or blood. Another eleven patients (16.2%) were agitated and needed general anaesthesia with intubation in order to protect themselves or for carrying out surgical procedures. Three patients (4.4%) also needed general anaesthesia with intubation for surgical procedures. Of these patients, one patient (1.5%) was admitted for a dislocated jaw after he had vomited because of alcohol intoxication. Another patient had a traumatic luxation of the elbow after a bicycle accident. The third patient had a hip luxation after a car accident. For reposition of the dislocations, these three patients were anaesthetised and needed endotracheal intubation at the ED.

None of the initially non-intubated patients needed intubation during the stay in the ED and nobody died while in the ED.

### Subgroup analysis of patients with GCS < 9 and without clinical traumatic brain injury with respect to patient characteristics and reasons for intubation

The results of the subgroup analysis of patients without traumatic brain injury and GCS < 9 are shown in Table 4 (*n* = 145). In this cohort, 35 patients (24.1%) needed intubation.

**Table 4:** Consultation and intoxication characteristics of intubated and non-intubated alcohol-intoxicated patients with GCS < 9 but without clinical traumatic brain injury, *n* = 145

	Total (n = 145)		Intubation (*n* = 35)		No Intubation (*n* = 110)		p
Sex, [n (%)]							
Male	94	(64.8)	22	(62.9)	72	(65.5)	0.779
Age, median (IQR)]	35.0	(22–48)	41.0	(25–48)	32.0	(22–47)	0.235
Type of admission, [n (%)]							
Ambulance	114	(79.2)	24	(70.6)	90	(81.8)	
External Hospital	6	(4.2)	5	(14.7)	1	(0.9)	
Police	1	(0.7)	0	(0.0)	1	(0.9)	
Air Rescue	7	(4.9)	5	(14.7)	2	(1.8)	
Walk-In	16	(11.1)	0	(0.0)	16	(14.5)	< 0.001
Triage, [n (%)]							
Life-threatening	37	(25.5)	21	(60.0)	16	(14.5)	
Urgent conditions	68	(46.9)	10	(28.6)	58	(52.7)	
Semi-urgent conditions	31	(21.4)	2	(5.7)	29	(26.4)	
Non-urgent conditions	2	(1.4)	0	(0.0)	2	(1.8)	
Missing	7	(4.8)	2	(5.7)	5	(4.5)	< 0.001
GCS, [median (IQR)]	6.0	(3–8)	3.0	(3–6)	6.5	(5–8)	< 0.001
Primary shock room use, [n (%)]	55	(37.9)	32	(91.4)	23	(20.9)	< 0.001
Mixed intoxication, [n (%)]	67	(46.2)	20	(57.1)	47	(42.7)	0.136
Medical mixed intoxication, [n (%)]	42	(29.0)	16	(45.7)	26	(23.6)	0.012
Illicit drug mixed intoxication, [n (%)]	37	(25.5)	8	(22.9)	29	(26.4)	0.679
Blood alcohol concentration, g/kg, [median (IQR)]^a^	1.6	(1.2–2.2)	1.3	(0.8–2.4)	1.6	(1.3–2.2)	0.048
Trauma (but no evidence of traumatic brain injury), [n (%)]	19	(13.1)	9	(25.7)	10	(9.1)	0.011
Chronic alcohol consumption, [n (%)]	35	(24.1)	4	(11.4)	31	(28.2)	0.044
Suicidal intent, [n (%)]	12	(8.3)	8	(22.9)	4	(3.6)	< 0.001
Fracture, [n (%)]	11	(7.6)	8	(22.9)	3	(2.7)	< 0.001
Bleeding, [n (%)]	1	(0.7)	0	(0.0)	1	(0.9)	0.571
Cerebral bleeding (spontaneous), [n (%)]	1	(0.7)	0	(0.0)	1	(0.9)	0.571
Superficial wound, [n (%)]	5	(3.4)	3	(8.6)	2	(1.8)	0.057
Contusion, [n (%)]	5	(3.4)	2	(5.7)	3	(2.7)	0.399
Luxation, [n (%)]	1	(0.7)	1	(2.9)	0	(0.0)	0.075
Aggression, [n (%)]	8	(5.5)	1	(2.9)	7	(6.4)	0.429
Psychiatrist involvement, [n (%)]	17	(11.7)	0	(0.0)	17	(15.5)	0.013
Sutures, [n (%)]	6	(4.1)	2	(5.7)	4	(3.6)	0.591
Police attendance, [n (%)]	18	(12.4)	3	(8.6)	15	(13.6)	0.429
Emergency surgery, [n (%)]	3	(2.1)	2	(5.7)	1	(0.9)	0.082
Hospital admission, [n (%)]	58	(40.0)	34	(97.1)	24	(21.8)	< 0.001

^**a**^data available in 90.3% (*n* = 131) of the patients

Similarly to the whole study population described above, there was no significant difference in age or gender between the intubated and non-intubated patients (*p* = 0.235 and *p* = 0.779, respectively). The main reason for intubation was reduced GCS (*n* = 19, 54.3%), followed by agitation/pain (*n* = 5, 14.3%), failure to ventilate/oxygenate (*n* = 4, 11.4%), and intubation to secure the airway (n = 4, 11.4%). Three patients suffered cardiac arrest; two of these patients were intubated in the prehospital phase.

In total 110 (75.9%) of all alcohol intoxicated patients without traumatic brain injury and a GCS < 9 had no secured airway. One of the non-intubated patients died from acute on chronic non-traumatic subdural hematoma and was not intubated because the medical team decided against further curative therapy.

Twelve of the non-intubated patients were treated at the ICU. One of those patients was intubated during the ICU phase because of missing protective airway reflexes. All patients were discharged from the ICU without complications.

## Discussion

Intubation in patients with alcohol intoxication is rare and the majority of intoxicated patients with GCS < 9 in our study were not intubated, without further serious complications. Predictors of intubations were occasional alcohol abuse and a history of trauma, independent of clinical evidence or history of traumatic brain injury. Although higher blood alcohol concentrations were not associated with intubation, special caution is needed in patients with concomitant trauma or other additional intoxications or diseases.

### Proportional incidence of intubation in alcohol intoxication

The proportional incidence of intubation is low (2.3% of all alcohol-intoxications presenting to our ED). This is lower than the intubation rate of 3.5% for drug intoxication found in the United States [18]. In addition to this, only about a fifth of patients with a GCS < 9 - that would mandate invasive airway protection in non-intoxicated trauma patients - were intubated in our study. This restrictive practice of invasive airway intervention may be a viable and safe approach, as our study did not reveal any severe complications (e.g. mortality or secondary intubations during ED monitoring). In order to better understand this practice and to identify patients at special risk, a detailed understanding of the clinical characteristics of intubated and non-intubated patients is important.

Two thirds of the performed intubations were carried out in the ED while one third was performed by ambulance services in the preclinical setting. It is reassuring that all patients were intubated in the initial evaluation process at admission and no emergency intubation during ED surveillance was necessary. This shows that the triage process using the Swiss triage scale together with the initial evaluation of physicians was adequate. Nevertheless, close monitoring of vital signs of any intoxicated patient is mandatory.

### Identifying patients at risk for an invasive airway management

In our study-population, the blood alcohol concentration measured on admission was lower in the group of intubated patients than in the non-intubated group and may therefore not be helpful as a sole criterion to identify patients at risk for intubation. A reason for this may also be that patients with chronical alcohol abuse, who may be used to higher alcohol concentrations, were intubated less often than occasional drinkers. The vulnerable group of chronic drinkers has nevertheless to be treated with caution, in order not to miss dangerous injuries or illnesses that overlay the frequently recognised chronic alcohol problem. Another patient group that warrants an increased level of suspicion were patients with medication intoxication in addition to alcohol alone - who were intubated more often. Especially patients with occasional alcohol consumption are at higher risk of trauma than chronic high volume drinkers [19]. Nearly half of the injuries presenting to EDs worldwide may be alcohol related according to the WHO [20]. In our study, patients with a history of trauma in general were intubated significantly more often than patients without a history of trauma. Half of all intubations in our group were trauma related. This was similar for patients with or without history or clinical evidence of a traumatic brain injury.

This is consistent with previous studies indicating that blood alcohol alters the primary assessment of trauma patients, resulting in a higher number of CT scans and a higher risk of intubation [21]. Previous research showed that patients with traumatic brain injury and alcohol intoxication had higher odds of being scored with a lower GCS [22]. Another possible reason for the significantly higher number of intubations in the group of trauma patients is the need for further imaging or immobilisation [21].

One of the key tasks of an emergency physician in the treatment of severely intoxicated patients is the identification of further diseases, other substances or generally other influences that may imitate or aggravate the symptoms of intoxication. This was also demonstrated in our study, in which, in addition to alcohol intoxication, patients with GCS < 9 often had other influencing factors that an emergency physician must consider when deciding for or against intubation.

### Reasons for intubation for all patients

Consideration of the detailed documented reasons for intubation indicates that the most common reason for intubation was a reduced GCS < 9 without other documented reasons. This is an indication that GCS is used even in intoxicated patients and non-trauma situations because of the lack of alternatives. The second most common reasons for intubation were preclinical problems with ventilation/oxygenation or traumatic brain injury with reduced GCS on ED admission. The scientific discussion about the role of alcohol in patients with traumatic brain injury is still ongoing and any influence on the outcome is still unclear. Even a protective effect of alcohol with unclear mechanism after traumatic brain injury has been discussed [23, 24]. The reason for this postulated positive effect is unclear. A link with intubation and oxygenation as well as ventilation, which is specifically crucial in traumatic brain injury patients, may hypothesized.

### Reasons for intubation in patients with GCS < 9 but without evidence of traumatic brain injury

Of special interest is the subgroup of patients without any evidence of traumatic brain injury but GCS < 9 at admission. Although the cut-off of GCS < 9 did not trigger intubation in this subgroup (intubation rate: 31.8%), the GCS in the intubated group was significantly lower than for non-intubated patients. The role of immobilisation and imaging as trigger for intubation in trauma without traumatic brain injury is unclear.

The other identified associations were similar in this group to the whole population.

### Limitations

As with all retrospective data analyses, we cannot rule out documentation bias or missed patients, despite careful data extraction and analysis. For instance, due to incomplete documentation, the GCS could not be broken down into its components to analysis GCS reduction in detail. However, these biases would affect all included patient groups and therefore probably do not influence our results. As these study results reflect the practice at one single centre in Switzerland, they may not be generalisable to all other settings, but just apply to treatment in our setting. Further prospective multicentre evaluation is needed.

No follow-up after discharge from hospital was possible with our retrospective data.

Due to the retrospective nature of the study, the weighting of alcohol intoxication against other additional factors affecting consciousness could not be fully clarified. Further prospective research is needed on this topic.

## Conclusions

Intubation in alcohol-intoxicated patients is rare and amongst intoxicated patients with GCS < 9, more than two thirds were not intubated in our study, but without severe complications. Trauma in general, independent of the history of a TBI, and a missing history of chronical alcohol abuse are associated with intubation, but not blood alcohol concentration. Special caution is required for intoxicated patients with trauma or other additional intoxications or diseases.

## Supplementary information

**Additional file 1 **: **Table S1.** Validation of the diagnosis or drug parser based on agreement with 500 manually coded ED reports.

## Data Availability

The datasets used and/or analysed during the current study are available from the corresponding author on reasonable request.
